# Mechanistic Understanding of Tyrosinase Inhibition by Polymeric Proanthocyanidins from *Acacia confusa* Stem Bark and Their Effect on the Browning Resistance of Fresh-Cut Asparagus Lettuce

**DOI:** 10.3390/molecules28083435

**Published:** 2023-04-13

**Authors:** Guanghui Li, Yaying Zhao, Zeya Qin, Shudong Wei, Dandan Liang, Yun Liang, Wei Song, Baomiao Ding

**Affiliations:** 1 College of Life Science, Yangtze University, Jingzhou 434025, China; 2 College of Life Science and Engineering, Henan University of Urban Construction, Pingdingshan 467036, China; 3 Henan Key Laboratory of Water Pollution Control and Rehabilitation Technology, Henan University of Urban Construction, Pingdingshan 467036, China

**Keywords:** *Acacia confusa*, proanthocyanidins, tyrosinase inhibition, conformational change, fresh-cut asparagus lettuce

## Abstract

Tyrosinase inhibitors are capable of preventing unfavorable enzymatic browning of fruits and vegetables. In this study, the capacity of *Acacia confusa* stem bark proanthocyanidins (ASBPs) to inhibit tyrosinase activity was evaluated. ASBPs were shown to be a high-potential inhibitor of tyrosinase with IC_50_ values of 92.49 ± 4.70 and 61.74 ± 8.93 μg/mL when using L-tyrosine and L-DOPA as the substrate, respectively. The structural elucidation performed with UV-vis, FT-IR spectroscopy, ESI-MS and thiolysis coupled to HPLC-ESI-MS suggested that ASBPs had structural heterogeneity in monomer units and interflavan linkages and consisted mainly of procyanidins dominant with B-type linkages. To gain insights into the inhibitory mechanisms of ASBPs against tyrosinase, different spectroscopic and molecular docking methods were further conducted. Results validated that ASBPs possessed the ability to chelate copper ions and could prevent the oxidation process of substrates by tyrosinase. The hydrogen bond formed with Lys-376 residue played a key role in the binding force of ASBPs with tyrosinase that induced a certain alteration in the microenvironment and secondary structure of tyrosinase, resulting in the enzymatic activity being ultimately restricted. It was also observed that ASBPs treatment effectively inhibited the activities of PPO and POD to retard the surface browning of fresh-cut asparagus lettuce and thus extended their shelf-life. The results provided preliminary evidence supporting the exploitation of ASBPs into potential antibrowning agents for the fresh-cut food industry.

## 1. Introduction

Fresh-cut fruit and vegetables are in high demand worldwide due to convenience and nutritional value [1]. However, these products are prone to enzymatic browning because of physical damage during processing which results in unfavorable effects on their quality and marketability [2]. Enzymatic browning is a process in which tyrosinase hydroxylates monophenols to *o*-diphenols, then oxidizes *o*-diphenols to *o*-quinones, and finally converts into melanin [3]. The control of the catalytic activity of tyrosinase has become a feasible way to prevent browning in the food industry [4]. Nowadays, more attention has been paid to discovering novel tyrosinase inhibitors in natural products with minor side effects. As previously reported, proanthocyanidins a group of natural polyphenol substances widely available in plant-derived foods and agricultural by-products were shown to display potent inhibitory activity against tyrosinase and melanogenesis [5,6,7], implying that they can be considered as a novel source of tyrosinase inhibitors and antibrowning agents for food preservation.

**Figure 1:**
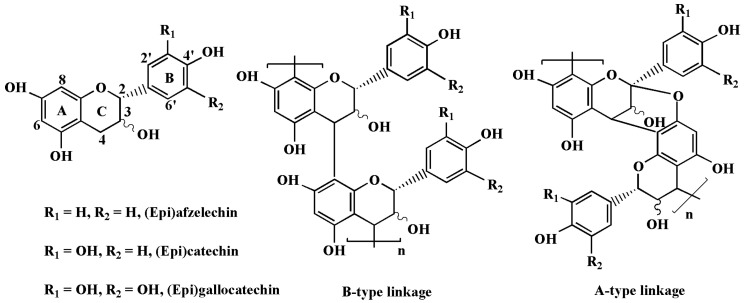
Chemical structure of flavan-3-ol monomer units and proanthocyanidins.

Proanthocyanidins also referred to as condensed tannins, are oligomers and polymers of flavan-3-ols linked principally through C-C bonds (B-type), which can occasionally coexist with an additional C-O-C ether bond (A-type) (Figure 1). Procyanidins with (epi)catechin as the exclusively constitutive units is the most abundant type proanthocyanidins in plants. The less common proanthocyanidins are propelargonidins and prodelphinidins consisting respectively of (epi)afzelechin and (epi)gallocatechin that are usually mixed with procyanidins [8]. Proanthocyanidins have been recognized to provide broad benefits to human health due to their polyphenolic structure [7,9,10,11,12].

*Acacia confusa* Merr., an indigenous species of Taiwan, is traditionally used as a medicinal plant [13]. Previous studies have shown that the crude extracts from leaves, twigs and flowers of *A*. *confuse* contained a wide variety of phenolic compounds [14,15,16]. Wei et al. [17] also found that *A*. *confusa* stem bark is rich in proanthocyanidins (ASBPs) and showed good DPPH radical scavenging activity and ferric reducing antioxidant power, more potent than those of the positive control, butylated hydroxyanisole (BHA). Since antioxidant agents can inhibit food browning and melanin formation in melanoma cells [2,18], it is reasonable to believe that the potent antioxidant activity of ASBPs also contributes to their antityrosinase and antibrowning potency. On this basis, the aims of this study were to evaluate the potential of ASBPs in terms of their inhibitory activity against tyrosinase as well as to investigate the inhibitory kinetics and the associated molecular mechanism via an integrated approach including UV-scanning, copper-ion chelation, fluorescence, circular dichroism (CD) spectroscopy and molecular docking simulation. A fresh-cut asparagus lettuce slice model was chosen to further confirm the antibrowning effect of ASBPs in real food preservation. Additionally, the structural elucidation of ASBPs was performed with UV-vis, FT-IR spectroscopy, ESI-MS and thiolysis coupled to reversed-phase HPLC-ESI-MS.

## 2. Results and Discussion

### 2.1. UV-vis Spectra of ASBPs

**Figure 2:**
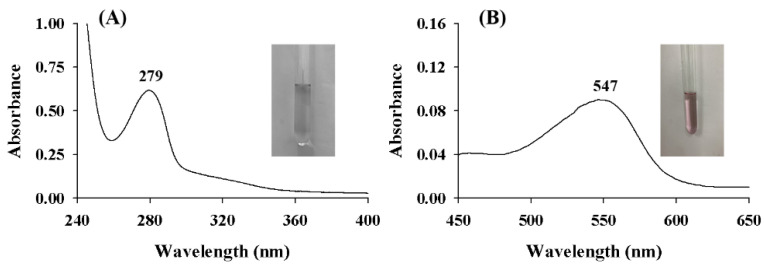
(**A**) UV-vis spectra of ASBPs and (**B**) their alcoholytic products. The inset images denote the colors of the samples before and after alcoholysis.

The UV-vis spectra of ASBPs and their alcoholytic products after butanol-HCl reaction are described in Figure 2. It was evident that there was a pronounced and symmetrical peak near 280 nm (Figure 2A), a typical characteristic of proanthocyanidins [19].

In a previous study, Xie and Dixon [20] revealed that the extension units of proanthocyanidins can be converted to colored anthocyanidins via alcoholysis under an acidic condition. The type of anthocyanidins attained is influenced by the type of proanthocyanidins being assayed [21]. If the alcoholytic product is cyanidin, the wavelength of maximum absorbance should be in the range of 540 to 550 nm, while if the alcoholytic product is delphinidin, then it should be in the range of 550 to 560 nm [22]. As shown in Figure 2B, the maximum absorption peak of ASBPs after alcoholysis appeared at 547 nm. It was inferred that ASBPs were primarily procyanidins and not prodelphinidins.

### 2.2. FT-IR Spectrum of ASBPs

**Figure 3:**
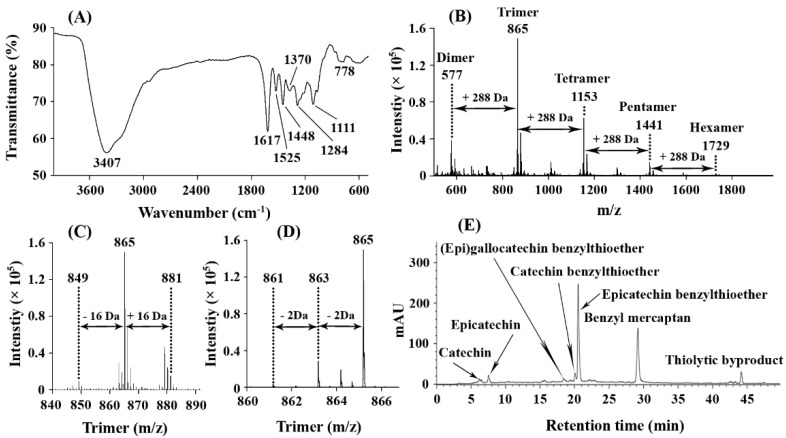
(**A**) FT-IR spectrum of ASBPs; (**B**) Negative-ion ESI-MS spectrum of ASBPs; (**C**,**D**) Amplified spectra of the trimer; (**E**) Reversed-phase HPLC chromatogram of thiolytic products of ASBPs.

Figure 3A demonstrates the results from FT-IR spectroscopy performed on the ASBPs. Specifically, the FT-IR spectrum displayed a broad and strong band at 3407 cm^−1^ which represented the -OH stretch vibration in the phenolic structure of ASBPs. In the 1617–1448 cm^−1^ region, the C=C stretching vibration of the aromatic ring was characterized [23]. The weak band at 1370 cm^−1^ was allocated to the -OH bending vibration and C-O stretching vibration. The peaks at 1284 and 1111 cm^−1^ were produced by the asymmetric stretching vibration of the heterocyclic C-ring ether bond. It has been claimed that the characteristic double peak at about 1520 cm^−1^ is only apparent when the proportion of prodelphinidins accounts for more than 60% in proanthocyanidins [24]. A single peak at 1525 cm^−1^ strongly suggested that ASBPs were principally comprised of procyanidins. Moreover, the report of Liu et al. [25] pointed out that there is one absorption band near 730 cm^−1^ when proanthocyanidins consist essentially of prodelphinidins, whereas one absorption band appears between 770 and 780 cm^−1^ when proanthocyanidins contain chiefly procyanidins. Figure 3A shows that a band at 778 cm^−1^ was clearly detected, further verifying that procyanidins were more predominant in ASBPs.

### 2.3. ESI Mass Spectrum of ASBPs

As can be seen in Figure 3B, the ESI mass spectrum of ASBPs recorded in the negative-ion mode exhibited a series of abundant ions with distances of 288 Da from dimer to hexamer (*m/z* 577, 865, 1153, 1441 and 1729). The difference between the major peaks was consistent with the mass of (epi)catechin, inferring that prolongation of ASBPs was due to the addition of (epi)catechin monomers. In the amplified spectrum of the trimer (Figure 3C), it was found that the major peaks were accompanied by a signal smaller than or larger than 16 Da. These less-intense signals were attributed to the heterogeneity of flavan-3-ol subunits [26], which in turn indicated the coexistence of (epi)afzelechin and (epi)gallocatechin in ASBPs. Furthermore, the minor ions with a 2 Da difference following the highest peaks were detected as well (Figure 3D). Proanthocyanidins with one A-type linkage can be readily identified on MS by their *m/z* of [M − H]^−^ being 2 Da less than that of B-type proanthocyanidins due to loss of two hydrogen atoms in formation of the interflavan ether bond [27]. To our knowledge, this was the first mass spectrometric evidence confirming the coexistence of A-type linkages in ASBPs but with less abundance. ESI-MS indicated that ASBPs had structural heterogeneity in monomer units and interflavan linkages and predominantly contained B-type procyanidins with a trace amount of propelargonidins and prodelphinidins.

### 2.4. Thiolysis of ASBPs for HPLC-ESI-MS Analysis

Figure 3E shows the reversed-phase HPLC chromatogram of thiolytic products of ASBPs. Based on the retention time of standard compounds and LC-MS data, epicatechin benzylthioether ([M − H]^−^ at *m/z* 411 with a fragment ion at *m/z* 287), (epi)gallocatechin benzylthioether ([M − H]^−^ at *m/z* 427 with a fragment ion at *m/z* 303) and catechin benzylthioether ([M − H]^−^ at *m/z* 411 with a fragment ion at *m/z* 287) were identified to be extension units, whereas catechin ([M − H]^−^ at *m/z* 289) and epicatechin ([M − H]^−^ at *m/z* 289) were identified as terminal units in ASBPs. The other two peaks observed were the excess of benzyl mercaptan and thiolytic byproduct. It could be inferred from the HPLC peak intensity that ASBPs essentially consisted of epicatechin units with catechin and (epi)gallocatechin present in a low proportion. LeRoux et al. [28] reported that the A-type linkage in proanthocyanidins is not susceptible to thiolysis, with unique thiolytic products representing A-linked dimers being occasionally detected. Compared with the data from ESI mass spectrum, however, no direct evidence for the A-type dimer or its benzylthioether was obtained in the thiolytic products from ASBPs. This might be because the amount of A-type linkage in ASBPs was less abundant, resulting in an A-linked product concentration below the detection limit. According to Chen et al. [12], the mean degree of polymerization (mDP) of ASBPs was estimated to be 8.1.

### 2.5. Inhibition of ASBPs on the Monophenolase Activity of Tyrosinase

**Figure 4:**
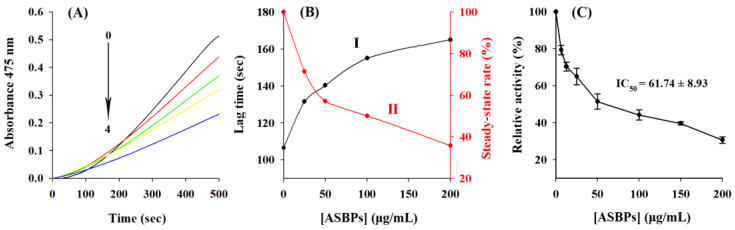
(**A**) Progress curves for the inhibition of monophenolase activity in tyrosinase by ASBPs; Effect of ASBPs on the (**B**) lag time and steady-state rate of monophenolase activity and (**C**) diphenolase activity in tyrosinase. Curves 0–4 represent ASBPs concentrations of 0, 25, 50, 100 and 200 µg/mL, respectively.

Figure 4A depicts the effect of ASBPs on the kinetic behavior of L-tyrosine oxidation via the monophenolase of tyrosinase. As can be observed from the results, the reaction system achieved a steady state after the lag time. Thereafter, the accumulation of the oxidation product was linear with increasing reaction time. Results shown in Figure 4B (curve I) demonstrate that the lag time of the enzyme increased rapidly at first and then more slowly. This can likely be ascribed to the combination of ASBPs with the *met* form of tyrosinase consequently prolonging the hydroxylation process of L-tyrosine [29].

It was also found from Figure 4B (curve II) that the steady-state rate decreased progressively and dose dependently with the gradual addition of ASBPs. When the ASBPs concentration was 200 μg/mL, the lag time extended from the initial 106.5 to 165 s, and the steady-state rate dropped by 64.3% of the total activity. The IC_50_ value representing the concentration of ASBPs at which monophenolase activity was reduced by 50% was measured to be 92.49 ± 4.70 μg/mL. The effective inhibition of monophenolase by ASBPs might arise from their structural feature of a benzene ring and many hydroxyl groups which was similar to that of L-tyrosine, a natural substrate of tyrosinase [5]. The results demonstrated that ASBPs could inhibit the hydroxylation of L-tyrosine by lengthening the lag time and reducing the steady-state rate of reaction.

### 2.6. Inhibition of ASBPs on the Diphenolase Activity of Tyrosinase

**Figure 5:**
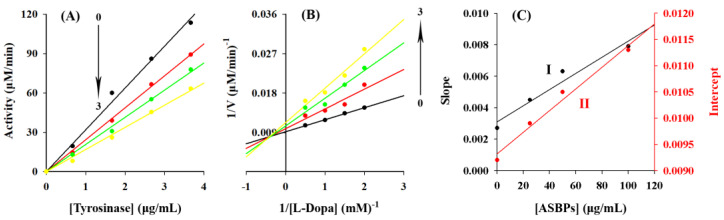
(**A**) Determination of the inhibitory mechanism of ASBPs on diphenolase in tyrosinase; (**B**) Lineweaver-Burk plots for inhibition of ASBPs on tyrosinase for the catalysis of L-DOPA; (**C**) Secondary plots of slope and intercept versus the concentration of ASBPs. Curves 0–3 represent ASBPs concentrations of 0, 25, 50 and 100 µg/mL, respectively.

The inhibition of diphenolase activity of tyrosinase by ASBPs was explored using L-DOPA as the substrate, the results of which are presented in Figure 4C. It could be seen that the activity of diphenolase was remarkably suppressed by ASBPs in a dose-dependent manner with an IC_50_ value of 61.74 ± 8.93 μg/mL. Compared with arbutin, a commercially available tyrosinase inhibitor, whose IC_50_ of diphenolase is 1387.2 μg/mL [12], ASBPs showed a better inhibitory effect. The reversibility of inhibition of ASBPs on diphenolase was assessed by plotting the enzyme concentrations versus residual enzyme activity in the presence of different concentrations of ASBPs (Figure 5A). It was obvious that all the lines showed good linearity and almost passed through the origin, and their slopes descended gradually as the concentrations of ASBPs increased from 0 to 100 µg/mL. This meant that ASBPs reversibly inhibited tyrosinase diphenolase activity and probably combined with tyrosinase through noncovalent interaction.

Inhibition type of ASBPs on diphenolase during the oxidation of L-DOPA was validated using the double-reciprocal Lineweaver-Burk plot. As displayed in Figure 5B, a series of straight lines with different slopes were intersected at the second quadrant close to the Y-axis, and the K_m_ values increased with the increase in ASBPs concentration, whereas an opposite trend was observed on the values of V_m_. These phenomena suggested that ASBPs exhibited mixed inhibition against diphenolase, with competitive inhibition being dominant [30], suggesting that ASBPs could not only bind to the active site of the tyrosinase competing with L-DOPA during catalysis but also bind with the tyrosinase-DOPA complex in a noncompetitive mode [31].

According to Figure 5C, the inhibition constants K_I_ and K_IS_ binding to the free enzyme and enzyme-substrate complex were 60 and 465 µg/mL for ASBPs, respectively. The K_IS_ value was 7.75 times larger than that of K_I_, indicating that the superior affinity of ASBPs for free enzyme over the enzyme-substrate complexes [32]. Overall, ASBPs could be considered to be an efficient, reversible and mixed-type inhibitor of the diphenolase of tyrosinase.

### 2.7. Inhibitory Effect of ASBPs on the Oxidation Process of L-Tyrosine and L-DOPA

**Figure 6:**
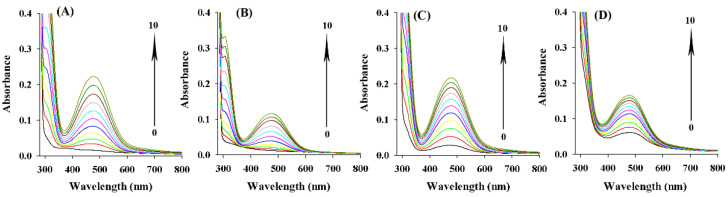
UV-vis spectra of the oxidation of L-tyrosine and L-DOPA by tyrosinase without (**A**,**C**) and with (**B**,**D**) ASBPs. Curves 0–10 represent 0–10 min after the addition of tyrosinase.

The UV-vis spectra of the oxidation of L-tyrosine and L-DOPA catalyzed by tyrosinase are given in Figure 6A–D. The peak at 475 nm was attributed to the generation of dopachrome [33]. Clearly, the spectra of oxidation products of L-tyrosine and L-DOPA without (Figure 6A,C) and with (Figure 6B,D) ASBPs exhibited a similar intensive absorption at 475 nm, but the peak intensity was different. After 10 min, the peak intensity in the presence of ASBPs reduced by 48.8% when L-tyrosine was selected as the substrate. Similarly, the oxidation of L-DOPA inhibited by ABSPs was 23.9% after the addition of tyrosinase for 10 min. The described observations suggested that ASBPs could bring down the formation of dopachrome by preventing the process of enzymatic oxidation of substrates, eventually leading to a reduction in the catalytic activity of tyrosinase.

### 2.8. Copper-Ion Chelating Ability

**Figure 7:**
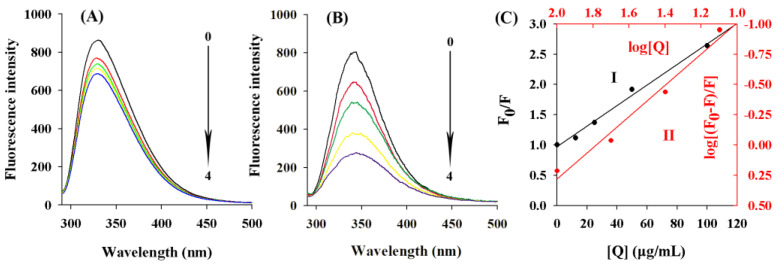
(**A**) Fluorescence spectra of the interaction between copper ion and ASBPs. Curves 0–4 represent copper ion concentrations of 0, 24, 48, 72 and 120 µM, respectively; (**B**) Fluorescence spectra of tyrosinase at different concentrations of ASBPs. Curves 0–4 represent ASBPs concentrations of 0, 12.5, 25, 50 and 100 µg/mL, respectively; (**C**) Plots of Stem-Volmer and log[(F_0_−F)/F] versus log[Q] for the quenching effect of ASBPs against tyrosinase.

Tyrosinase is a copper-containing polyphenol oxidase, and the domain of tyrosinase contains a highly conserved binuclear copper binding site [34]. It is widely accepted that the inhibition of the metalloenzyme depends on the possibility for the inhibitor to chelate the metal ion(s) in the active site [21]. The fluorescence spectra of ASBPs treated with various concentrations of copper ion were recorded to study their interactions. As shown in Figure 7A, with the addition of copper ion in sequence, a gradual decrease in the fluorescence emission intensity of ASBPs was observed, which provided direct evidence for the interaction between ASBPs and the copper ion as well as the formation of the ASBPs-Cu complex [6]. It could be deduced that the inhibition of tyrosinase by ASBPs might be caused by chelating copper ions of this enzyme.

### 2.9. Fluorescence Quenching Analysis

The fluorescence emission spectra of tyrosinase at different concentrations of ASBPs with the excitation wavelength at 280 nm are described in Figure 7B. Obviously, the addition of ASBPs led to a distinct decline in tyrosinase fluorescence strength, indicating that the complex of ASBPs with tyrosinase was formed and quenched its intrinsic fluorescence. Additionally, the fluorescence quenching induced by ASBPs was accompanied by a slight red-shift of the maximum emission wavelength from 342 to 344 nm. According to Chen et al. [21], this shift is an indication that ASBPs can interact with the binding active site of tyrosinase and induce the fluorophore residues in tyrosinase tending to be less hydrophobic in the microenvironment.

The Stern-Volmer plot of the fluorescence quenching of tyrosinase by ASBPs is presented in Figure 7C. A good linearity was found between F_0_/F and [Q] (curve I), implying the occurrence of a singly type of quenching, either static or dynamic. As calculated in the Stern-Volmer equation, the values of K_sv_ and K_q_ were (1.08 ± 0.01) × 10^−2^ mL/µg and (1.08 ± 0.01) × 10^6^ mL/(µg·s), respectively. In consideration of the mean molecular weight of ASBPs (8.1 × 288 g/mol), the quenching rate constant K_q_ was further transformed into 2.51 × 10^12^ L/(mol·s). In previous studies, the maximum quenching constant for the scattered collisions was 2.0 × 10^10^ L/(mol·s) [35], much lower than the K_q_ value of the tyrosinase quenching procedure initiated by ASBPs, which indicated that the static quenching dominated in the quenching process of tyrosinase by ASBPs. From the plot of log[(F_0_-F)/F] versus log[Q] (curve II), the value of n was determined to be 1.06 ± 0.02. This was indicative of just one single binding site on tyrosinase for ASBPs.

### 2.10. Synchronous Fluorescence Spectra

**Figure 8:**
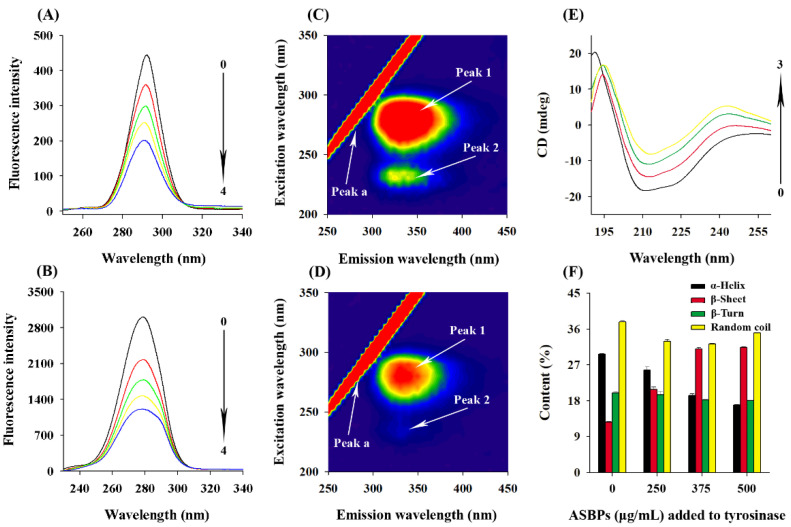
Synchronous fluorescence spectra of tyrosinase at different concentrations of ASBPs: (**A**) Δλ = 15 nm and (**B**) Δλ = 60 nm. Curves 0–4 represent ASBPs concentrations of 0, 10, 20, 30 and 40 µg/mL, respectively; Three-dimensional fluorescence spectra of tyrosinase without (**C**) and with (**D**) ASBPs; (**E**) Far-UV CD spectra of tyrosinase at different concentrations of ASBPs. Curves 0–3 represent ASBPs concentrations of 0, 250, 375 and 500 µg/mL, respectively; (**F**) Secondary structure changes of tyrosinase at different concentrations of ASBPs.

Figure 8A,B illustrate the synchronous fluorescence spectra of tyrosinase in the presence of ASBPs at the wavelength interval (Δλ) of 15 and 60 nm. The shift in position of emission maximum of the corresponding spectra reflects the changes in the microenvironment of the tyrosine and tryptophan residues in tyrosinase, respectively. Evidently, the fluorescence intensity of the two residues declined dramatically with the increase of ASBPs concentration, indicating that the both tyrosine and tryptophan residues of tyrosinase contributed importantly to the interaction with ASBPs [36].

Figure 8A shows that the maximum emission wavelength had a significant blue shift from 292.2 nm to 290.2 nm when Δλ was set to 15 nm, whereas that shown in Figure 8B kept the position at the investigated concentration range when Δλ was set to 60 nm. It was inferred from the results that the conformation of tyrosinase was somewhat changed and the hydrophobicity around tyrosine residues increased in the presence of ASBPs, making them less exposed to the solvent [37], while the microenvironment around tryptophan residues displayed no discernable change.

### 2.11. Three-Dimensional Fluorescence Spectra

The three-dimensional fluorescence spectra of tyrosinase without and with ASBPs are shown in Figure 8C,D. Peak a is the Rayleigh scattering peak (λ_ex_ = λ_em_), peak 1 (λ_ex_/λ_em_ = 280 nm/340 nm) is the spectral behavior of tyrosine and tryptophan residues and peak 2 (λ_ex_/λ_em_ = 230 nm/340 nm) mainly represents the fluorescent features of the polypeptide chain backbone of tyrosinase induced by the π→π* transition [38]. It was clear that the values of peak 1 reduced noticeably and peak 2 almost disappeared after the addition of ASBPs. These changes evidently proved that the interaction between ASBPs and tyrosinase might alter the microenvironment of tyrosine residue and cause the unfolding of polypeptides of tyrosinase [36], which supported the findings of fluorescence quenching and CD spectroscopy analysis.

### 2.12. CD Spectra

The conformational changes of tyrosinase induced by ASBPs were measured with CD spectroscopy. As seen in Figure 8E, two negative bands at 212 nm and 222 nm were observed in the far-UV CD spectrum of tyrosinase, which have been proven to be the characteristic peaks of α-helix structures [31]. When tyrosinase was treated by different concentrations of ASBPs (0, 250, 375 and 500 μg/mL), the intensities of CD spectra were reduced markedly without any significant shift in peak position. This indicated that the secondary structure content of α-helical declined, but it was still dominant in tyrosinase conformation after ASBPs binding. A similar phenomenon has been reported in the interaction of pentagalloylglucose with tyrosinase [32]. The contents of the secondary structures calculated via CDNN program are listed in Figure 8F. Compared with the free tyrosinase, it showed a decrease in α-helix contents from 29.67% to 16.87% and an increase in β-sheet contents from 12.63% to 31.37%. The results suggested that the combination of ASBPs with tyrosinase probably had destroyed its hydrogen bonding networks and given rise to a looser and unstable structure of the enzyme, which caused the activity of tyrosinase to be restricted [39].

### 2.13. Molecular Docking

**Figure 9:**
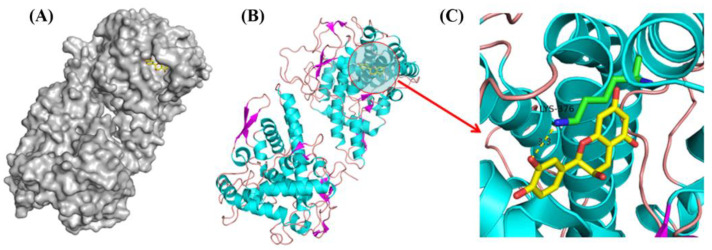
(**A**) Surface and (**B**) cartoon diagrams of the predicted binding mode of epicatechin, the main constitutive unit of ASBPs to tyrosinase; (**C**) Close-up of the interaction between epicatechin and tyrosinase.

To improve the understanding of the binding of ASBPs to tyrosinase, the main constitutive unit of ASBPs, i.e., epicatechin, was used to perform molecular docking. The predicted mode of binding between epicatechin and tyrosinase with the interaction energy of −7.20 kcal/mol is depicted in Figure 9A–C. As can be seen, epicatechin could penetrate into the active site of tyrosinase [34]. The phenolic hydroxyl on the benzene ring of epicatechin formed a hydrogen bond (3.3 Å) interaction with the Lys-376 residue of tyrosinase. Hydrogen bonding might be the dominant driving force involved in the formation of the ASBPs-tyrosinase complex. The molecular docking analysis provided a clear explanation for the binding mechanism of ASBPs with tyrosinase.

### 2.14. Antibrowning Effect of ASBPs

**Figure 10:**
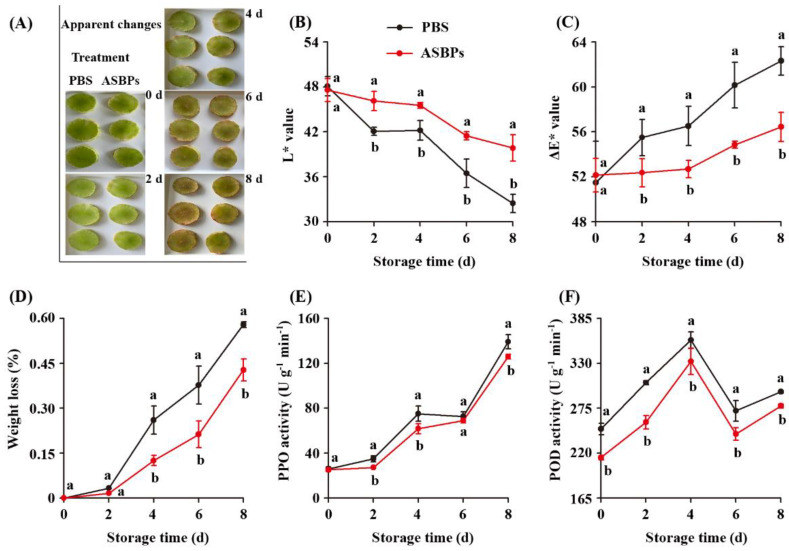
Effect of ASBPs treatment on the visual appearance (**A**), chromaticity L* (**B**), ∆E (**C**), wight loss (**D**), PPO (**E**) and POD (**F**) activities of fresh-cut asparagus lettuce slices during storage at 4 °C for 8 days. Different letters indicate significant differences between groups at each of the sampling timepoints (*p* < 0.05).

Fresh-cut asparagus lettuce slices were chosen to evaluate the antibrowning effect of ASBPs in real food preservation, as shown in Figure 10. It was intuitively observed that the asparagus lettuce slices treated with ASBPs could prevent the appearance loss to a certain extent after 8 days of storage at 4 °C as compared to control samples (Figure 10A). The L* and ∆E are the main parameters usually used to track the browning process of fresh-cut fruits and vegetables [40]. In Figure 10B,C the L* value of the fresh-cut asparagus lettuce slices gradually decreased while the ΔE value progressively increased with the extension of storage time, suggesting that the browning occurred in these samples. Nevertheless, the values of L* and ΔE in the ASBPs-treated slices respectively maintained lower and higher levels compared to those of the control slices. The above findings confirmed that ASBPs have potential as an antibrowning agent worthy of further development.

The changes in weight loss of fresh-cut asparagus lettuce slices with different treatments during storage were also investigated, and the results are presented in Figure 10D. Apparently, weight loss of all samples increased as the storage time prolonged. Compared with those of the control, the slices treated with ASBPs exhibited lower weight loss. This was presumably due to the inhibition of ASBPs against respiratory chain enzyme activity consequently reducing the respiratory of fresh-cut asparagus lettuce slices [41]. Polyphenol oxidase (PPO) and peroxidase (POD) are crucial enzymes closely related to the surface browning of fresh-cut fruit and vegetables [42]. Figure 10E,F show the effect of ASBPs on the PPO and POD activities of fresh-cut asparagus lettuce slices. The activity of PPO of slices, regardless of treatment, increased gradually during the entire storage period, while that of POD initially increased in the first 4 days and decreased later. The PPO and POD activities in the ASBPs-treated group were noticeably more inactivated compared to those of untreated group. The results illustrated that ASBPs could alleviate the surface browning and extend the shelf life of fresh-cut asparagus lettuce slices via the inhibition of the activities of PPO and POD [4].

## 3. Materials and Methods

### 3.1. Chemicals and Plant Materials

Mushroom tyrosinase, L-3,4-dihydroxyphenylalanine (L-DOPA), benzyl mercaptan, catechin and epicatechin were purchased from Sigma-Aldrich (St. Louis, MO, USA). L-Tyrosine and copper chloride (CuCl_2_) were supplied by Sangon Biotech Co., Ltd. (Shanghai, China). HPLC-grade acetonitrile and trifluoroacetic acid as well as other analytical-grade chemicals were acquired from Macklin (Shanghai, China). The stem barks of A. confusa were collected from Xiamen botanical garden (Xiamen, China), freeze-dried using a Detianyou FD-1 desktop freeze-dryer (Beijing, China) at −56 °C for 72 h, ground to powder and stored at −20 °C before the subsequent analysis.

### 3.2. Extraction and Separation of ASBPs

The ASBPs were extracted and separated using a procedure previously described by Chen et al. [12]. Specially, 25 g of ground sample was subjected to extraction thrice with 250 mL of 70% acetone solution for 24 h each time at room temperature and normal pressure. The extracts were centrifuged at 5000× *g* for 15 min and pooled, and the acetone was removed under reduced pressure. To remove pigments, lipids and other nonpolar compounds, the remaining aqueous fraction was partitioned with hexane and dichloromethane successively. Afterward, the aqueous layer containing crude proanthocyanidins was applied onto a 30 × 2.5 cm column of Sephadex LH-20 (GE Healthcare, Uppsala, Sweden), which was first washed with 50% methanol until the eluent turned colorless, which followed by eluting with 70% acetone. The collected acetone eluents were then rotary-evaporated and lyophilized to obtain the purified ASBPs.

### 3.3. Structural Elucidation of ASBPs

The structural profile of ASBPs was elucidated using UV-vis, FT-IR spectroscopy, ESI-MS and thiolysis coupled to HPLC-ESI-MS. UV-vis spectra of ASBPs and their alcoholytic products after butanol-HCl reaction were recorded at wavelengths of 240–400 nm and 400–800 nm, respectively, on a Purkinje TU-1900 spectrophotometer (Beijing, China). The procedure for butanol-HCl assay was adopted from Li et al. [43]. The FT-IR spectrum of ASBPs was detected with a Thermo Nicolet 470 infrared spectrometer (Madison, USA) in the wavenumber range of 500–4000 cm^−1^ with a resolution of 4 cm^−1^ [44]. ESI mass spectrum of ASBPs was measured using an AB Sciex TripleTOF 5600 mass spectrometer (Framingham, MA, USA) as detailed in Ren et al. [20]. Thiolysis of ASBPs with benzyl mercaptan for HPLC-ESI-MS analysis was conducted as described in our previous study [10]. Thiolytic product was injected into an Agilent 1200 system (Palo Alto, CA, USA) interfaced to the AB Sciex QTrap 3200 (Foster City, CA, USA) with a reversed phase 250 × 4.6 mm (5 µm, particle size) Hypersil ODS column (Elite, Dalian, China). The binary mobile phases consisted of A (0.5% trifluoroacetic acid in water, *v*/*v*) and B (acetonitrile), which were delivered in a linear gradient of B from 12 to 80% (*v*/*v*) in 45 min.

### 3.4. Effect of ASBPs on Tyrosinase Activity

The inhibition of ASBPs against the monophenolase (L-tyrosine as the substrate) and diphenolase (L-DOPA as the substrate) of tyrosinase was examined using our already established method [12]. In the 3 mL phosphate buffer (PBS, 50 mM, pH 6.8) assay system, 0.1 mL of ASBPs with different concentrations, 0.3 mL of 2 mM L-tyrosine (or 0.5 mM L-DOPA) and 0.05 mL of tyrosinase were successively added. Then, the growth curve of the absorbance of the mixture with time at the wavelength of 475 nm was detected with a UV-vis spectrophotometer (Purkinje TU-1900). In the study of monophenolase, steady-state rate and lag time were acquired by extrapolating the curve to the abscissa. Inhibition type and inhibition constants of ASBPs on diphenolase were detected based on the Lineweaver-Burk plot and secondary plots of the apparent K_m_/V_m_ or 1/V_m_ versus different ASBPs concentrations, respectively.

### 3.5. Effect of ASBPs on the Oxidation of L-Tyrosine and L-DOPA

The effect of ASBPs on the oxidation of L-tyrosine and L-DOPA by tyrosinase were evaluated with UV-vis spectrophotometry following the method of Cui et al. [45]. The reaction media contained 1.2 mM of L-tyrosine or 0.5 mM of L-DOPA, ASBPs (66.67 μg/mL), tyrosinase (5.33 μg/mL) and PBS (50 mM, pH 6.8). The time-dependent UV absorption spectra of the media were monitored at wavelengths from 260 to 800 nm using a Purkinje TU-1900 spectrophotometer.

### 3.6. Copper-Ion Chelation

The copper-ion chelating ability of ASBPs was determined with a fluorescence spectrophotometer (F-4600, Tokyo, Japan), with reference to a previous report [6]. In brief, 0.1 of mL copper chloride with different concentrations, 0.1 mL of ASBPs (60 μg/mL) and 2.8 mL of 50 mM PBS (pH 6.8) were mixed and incubated at 25 °C for 2 min. The mixture was measured in the 290–400 nm emission wavelength range at an excitation wavelength of 280 nm.

### 3.7. Fluorescence Spectroscopy

The fluorescence quenching of tyrosinase by ASBPs was studied according to Cui et al. [45]. In this assay, different concentrations of ASBPs were mixed with tyrosinase (1 mg/mL) and incubated at 303 K for 10 min. The fluorescence emission spectra of the resulting solution were recorded on a F-4600 fluorescence spectrophotometer with an excitation wavelength of 280 nm and emission wavelengths of 290–500 nm. The Stern-Volmer equation was employed to analyze the fluorescence data:

F_0_/F = 1 + K_sv_[Q] = 1 + K_q_τ_0_[Q] (1)

where F_0_ and F represent the fluorescence intensities of tyrosinase without and with ASBPs, respectively; and K_sv_, K_q_, τ_0_ and [Q] are the Stern-Volmer quenching constant, the quenching rate constant, the average lifetime of unquenched fluorophore (10^−8^ s) and the concentration of ASBPs, respectively.

For static quenching, the apparent binding constant (K_a_) and the number of binding sites (n) between ASBPs and tyrosinase could be estimated according to following the double-logarithmic equation:

log[(F_0_ − F)/F] = logK_a_ + nlog[Q] (2)

The synchronous fluorescence spectra of tyrosinase (0.4 mg/mL) without and with ASBPs were scanned by setting the excitation and emission wavelength intervals (Δλ) at 15 and 60 nm, respectively. The three-dimensional fluorescence spectra of tyrosinase (0.025 mg/mL) without and with ASBPs were collected at excitation and emission wavelengths from 200 to 350 nm and 250 to 450 nm, respectively.

### 3.8. Far-UV CD Spectroscopy

The CD spectra in the far-UV (190–250 nm) of tyrosinase treated with different concentrations of ASBPs were measured on a Bio-Logic MOS-500 spectrometer (Claix, France) under constant nitrogen flush. The concentration of tyrosinase was fixed at 0.3 mg/mL. The proportion of different secondary structures of tyrosinase was calculated with CDNN software [32].

### 3.9. Molecular Docking Study

Molecular docking was performed with AutoDock Vina (version 1.1.2) to explore the probable interaction of ASBPs with tyrosinase. Epicatechin, the main constitutive unit of ASBPs, was selected as the ligand. The structure of epicatechin was downloaded from the PubChem database and geometrically optimized in ChemBio3D Ultra 14.0. The crystal structure of *Agaricus bisporus* tyrosinase was obtained from the Protein Data Bank (PDB ID: 2Y9W) [34]. To avoid hindrance during docking, all water molecules were removed before docking, and polar hydrogen atoms and gasteiger charges were added to compute the pdbqt format. The optimal binding mode was chosen based on the docked conformation with the lowest free energy.

### 3.10. Color and Weight Loss Measurements

The changes of color of asparagus lettuce slices during storage was measured according to a reported method [46]. Asparagus lettuces were washed, peeled and manually sliced into a 3 mm thickness with a stainless-steel knife. The freshly prepared slices were immediately submerged in PBS (control, pH 6.8) or ASBPs (0.5 mg/mL) for 5 min and then drained. Subsequently, the samples were placed in polyethylene plastic bags and stored at 4 °C for 8 days. Color, expressed as L*, a* and b* values, was determined using a CR-400 Minolta colorimeter (Osaka, Japan). Total color difference (ΔE) was calculated as follows:

ΔE = [(L*_t_ − L*_initial_)^2^ + (a*_t_ − a*_initial_)^2^ + (b*_t_ − b*_initial_)^2^]^1/2^ (3)

During the 8 days of storge at 4 °C, the weight loss from samples was also monitored. The values are expressed as percent losses from the initial weights of the sample based on the following equation:

Weight loss (%) = (W_i_ − W_f_)/W_i_ × 100 (4)

where W_i_ corresponds to the initial weight, and W_f_ is the final weight at each storage day interval of asparagus lettuce.

### 3.11. PPO and POD Activity Measurements

The extraction and assay of PPO and POD activity were conducted as reported by Kim et al. [47], with minor modifications. Asparagus lettuce slices were homogenized in 50 mM of PBS containing polyvinylpyrrolidone (pH 6.8), and the filtrate was centrifuged at 5000× *g* for 10 min at 4 °C. The supernatant was collected as the crude enzyme extract. PPO activity: the reaction mixture contained 0.5 mL of enzyme extract, 1 mL of 0.02 M catechol and 1.5 mL of 50 mM PBS (pH 6.8). The absorbance was recorded every 30 s at 410 nm within 3 min. For POD activity, the assay was performed using 0.5 mL of enzyme extract, 1.0 mL of 0.3% H_2_O_2_, 1.0 mL of 0.2% guaiacol and 0.8 mL of 50 mM PBS (pH 6.8). The absorbance was read every 30 s at 470 nm within 3 min. One unit (U) of PPO and POD activity was defined as an increase in 0.01 absorbance per minute per gram fresh weight.

### 3.12. Statistical Analysis

Data are expressed as the mean ± standard deviation (SD). SPSS 17.0 (Chicago, IL, USA) was used to perform one-way analysis of variance (ANOVA). A *p* value less than 0.05 was defined as statistical significance.

## 4. Conclusions

This is the first detailed report on chemical elucidation of ASBPs that essentially consisted of B-type procyanidins with minor A-type linkages and an mDP of 8.1. Both monophenolase and diphenolase activities of tyrosinase were dose-dependently suppressed by ASBPs. The mechanisms by which ASBPs inhibited tyrosinase activity might be achieved via the complexation of copper ions preventing the formation of oxidation products of substrates L-tyrosine and L-DOPA as well as changing the microenvironment and secondary structure of tyrosinase. ASBPs were also found to be effective in delaying the development of surface browning and extending the shelf life of fresh-cut asparagus lettuce slices during storage. Collectively, these results suggested that ASBPs have the potential to be developed as a tyrosinase inhibitor able to counteract the enzymatic browning of fresh-cut fruits and vegetables.

## Data Availability

The datasets used and/or analyzed during the current study are available from the corresponding author upon reasonable request.

## Sample Availability

Samples of the compounds are not available from the authors.
